# Incidence Rate of Type 2 Diabetes Mellitus after Gestational Diabetes Mellitus: A Systematic Review and Meta-Analysis of 170,139 Women

**DOI:** 10.1155/2020/3076463

**Published:** 2020-04-27

**Authors:** Zhuyu Li, Yunjiu Cheng, Dongyu Wang, Haitian Chen, Hanqing Chen, Wai-kit Ming, Zilian Wang

**Affiliations:** ^1^Department of Obstetrics and Gynaecology, The First Affiliated Hospital of Sun Yat-sen University, Guangzhou, China; ^2^Department of Cardiology, The First Affiliated Hospital of Sun Yat-sen University, Guangzhou, China

## Abstract

**Objective:**

The reported incidence of type 2 diabetes mellitus (T2DM) after gestational diabetes (GDM) varies widely. The purpose of this meta-analysis was to define the incidence rate of T2DM among women with a history of GDM and to examine what might modulate the rate. *Research Design and Methods*. We searched PubMed and Embase for terms related to T2DM after GDM up to January 2019. Large cohort studies with sample size ≥300 and follow-up duration of at least one year were included. Data from selected studies were extracted, and meta-analysis was performed using the random-effects model. Subgroups analyses were based on the sample size of gestational diabetes, geographic region, maternal age, body-mass index, diagnostic criteria, and duration of follow-up.

**Results:**

Twenty-eight studies involving 170,139 women with GDM and 34,627 incident cases of T2DM were identified. The pooled incidence of T2DM after GDM was 26.20 (95% CI, 23.31 to 29.10) per 1000 person-years. Women from Asia and those with older age and higher body mass index seem to experience higher risk of developing T2DM. The incidence rate of T2DM was lowest when applying IADPSG (7.16 per 1000 person-years) to diagnose GDM. The risk of developing T2DM after GDM increased linearly with the duration of follow-up. The increments per year of follow-up were estimated at 9.6‰. The estimated risks for T2DM were 19.72% at 10 years, 29.36% at 20 years, 39.00% at 30 years, 48.64% at 40 years, and 58.27% at 50 years, respectively.

**Conclusions:**

The findings of very high incidence of T2DM after GDM add an important insight into the trajectory of the development of T2DM in the long-term postpartum periods, which could provide evidence for consultant and might motivate more women with GDM to screen for T2DM. This trial is registered with PROSPERO identifier CRD42019128980.

## 1. Introduction

Gestational diabetes mellitus (GDM) is a condition in which glucose intolerance is first recognized during pregnancy. After delivery, these affected women are advised to perform glucose tolerance test as to screen for type 2 diabetes mellitus (T2DM) [1]. However, the low rate of postpartum screening for T2DM implies that we should make more efforts to improve their compliance [2]. Postpartum follow-up screening is often a responsibility for obstetricians who often pay more attention to pregnancy-related diseases but might ignore the conversion of GDM to T2DM. Although meta-analysis has shown that women with GDM have at least a seven-fold increased risk of developing T2DM, compared with those who have had a normoglycaemic pregnancy [3], there is no available meta-analysis on the incidence of T2DM after GDM. In addition, the reported incidence of T2DM after GDM varied widely from 1.3% [4] to 70% [5]. Consequently, demonstrating the incidence of T2DM among women with a history of GDM may help obstetricians attach more importance to this conversion and thus encourage more affected women to screen for T2DM.

Therefore, we conducted a meta-analysis with the following aims: (1) to ascertain the incidence rate of T2DM after GDM; (2) to perform a subgroup analysis based on study characteristics, including study designs, geographic region, sample sizes, age, body mass index (BMI), GDM, and T2DM criteria; and (3) to explore the link between duration of follow-up and incidence of T2DM after GDM.

## 2. Materials and Methods

### 2.1. Data Sources and Searches

This meta-analysis follows the MOOSE guidelines. We performed a comprehensive search of prospective, retrospective, randomized, or cohort studies in the electronic databases MEDLINE (source PubMed, January 1, 1966, to January 1, 2019) and Embase (January 1, 1980, to January 1, 2019) using the following text and key words in combination both as MeSH terms and text words “gestational diabetes,” “diabetes mellitus,” “type 2 diabetes mellitus,” “NIDDM,” and “non-insulin dependent diabetes mellitus.” The search was limited to humans. We searched articles published in any language and scrutinized references from these studies to identify other relevant studies.

### 2.2. Study Selection

To be included in this meta-analysis, primary studies had to report the incidence rates of T2DM among the women with a history of GDM and the explicit follow-up duration. To enhance the representative and reliability, we made exclusion criteria as follows: (1) The amount of women with GDM in the study was less than 300. (2) The duration of follow-up was shorter than 12 months after the end of the index pregnancy. (3) There existed any form of intervention on preventing or delaying diabetes among women with gestational diabetes. (4) The study was a case series, letter, review, commentary, or editorial. For studies published in more than one report (duplicates), we considered the most comprehensive study that reported the largest sample size.

### 2.3. Data Extraction

Articles were reviewed and cross-checked independently by two authors (ZYL and YJC). The percentage agreement between the two authors on the quality review ranged from 89% to 100%. Any disagreements were resolved by consensus between the authors (ZYL and YJC). The relevant data extracted included the year of publication, first author, source country, geographic region (defined as Europe, North America, Asia, etc.), study design, baseline patient characteristics, the total number of women with GDM and those with T2DM, follow-up data, and diagnostic criteria for GDM and T2DM.

### 2.4. Data Synthesis and Analysis

The primary end point of the study was the incidence rate of T2DM after GDM expressed as per 1000 person-years of follow-up and is presented with 95% confidence intervals (CIs). The effect size was calculated using a random-effects model because we considered that the different patient groups in different countries during different periods were unlikely to have a common effect size. The mean duration of follow-up represented for the length of follow-up. Median length of follow-up could be used to estimate mean length because the sample size of each included study was larger than 25 according to the simulations derived from Hozo and colleagues [6].

Weighted meta-analytic prevalence estimates for outcomes were calculated with the variance-stabilizing Freeman-Tukey double-arcsine transformation with an inverse-variance random-effects model [7]. Heterogeneity was assessed with the *I*^2^ statistic [8], where *I*^2^ of at least 50% indicated significant heterogeneity. Sources of between-study heterogeneity were investigated using subgroup analysis.

We used a metaregression model to assess the relation between follow-up years and incidence rate of T2DM after GDM. We used Stata, version 14.0 (StataCorp) for all analyses. Statistical tests were two sided and used a significance level of *P* < 0.05.

## 3. Results

### 3.1. Study Selection

**Figure 1:**
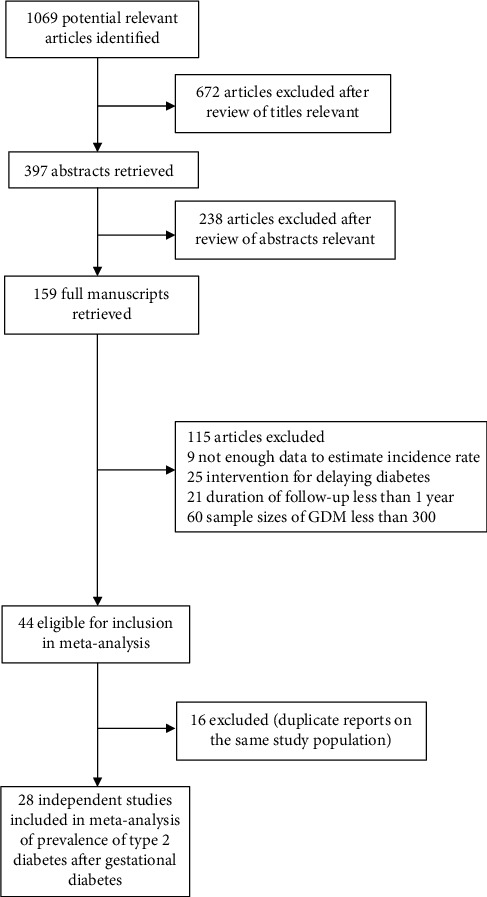
Flowchart of the selection of studies included in meta-analysis.

With the search strategy, 1069 unique citations were initially retrieved. Of these, 159 articles were considered of interest and full text was retrieved for detailed evaluation. One hundred and thirty-one of these 159 articles were subsequently excluded, and finally, 28 articles were included in the meta-analysis (Figure 1).

### 3.2. Study Characteristics

Twenty-eight independent studies [9–36] reporting 170,139 women with GDM with 1,879,062 person-years of follow-up and a total of 34,627 incident cases of T2DM were identified (Table 1). The mean sample size of the studies was 6076 (range from 304 to 56,884). Fourteen studies (*n* = 155,340) were retrospective investigations, and the other fourteen studies (*n* = 14,799) were prospective.

The mean (SD) duration of follow-up was 8.35 (6.43) years (range, 1 to 30 years; interquartile range, 3.86 to 11.35 years). Patients were followed up for an average of over three years in a majority of studies (78.57%).

Eleven studies were from Europe, seven from North America, six from Asia, two from Australasia, and two from intercontinental countries. Studies were published between January 1991 and September 2018.

### 3.3. Incidence of T2DM after GDM

**Figure 2:**
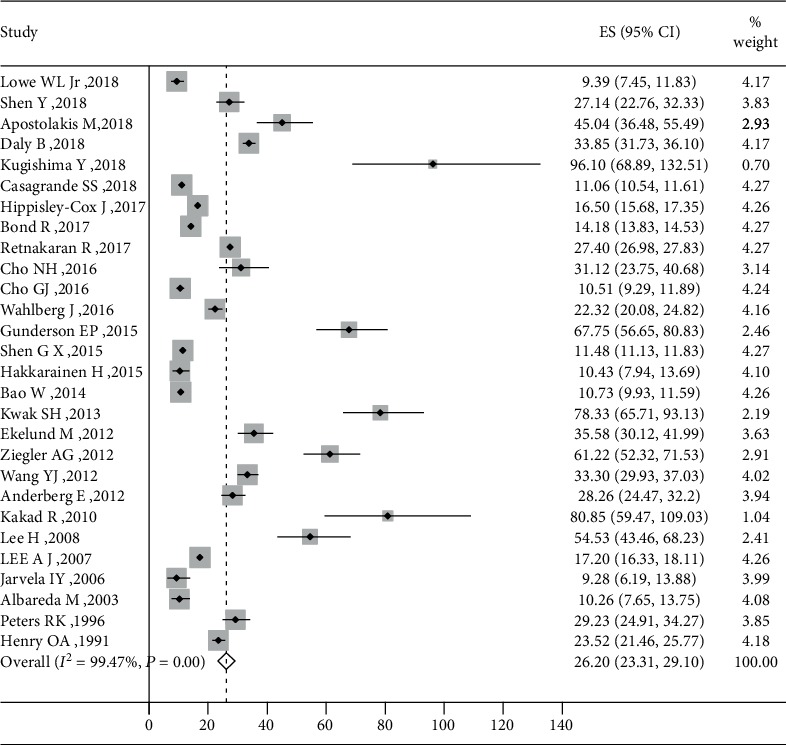


**Figure 3:**
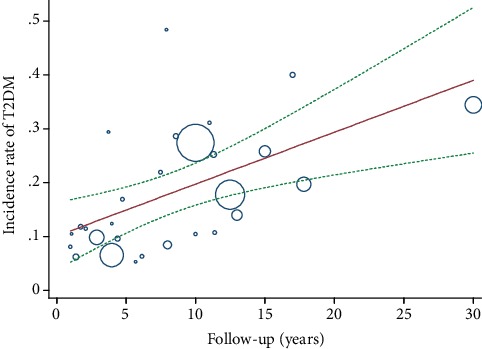


All identified studies reported an incidence rate meeting all eligibility criteria for inclusion in a meta-analysis. The incidence proportion estimates ranged from 9.28 [16] per 1000 person-years to 96.10 [34] per 1000 person-years. The overall incidence rate of T2DM after GDM was 26.20 (95% CI, 23.31 to 29.10) per 1000 person-years with very high between-sample heterogeneity (*P* < 0.001; *I*^2^, 99.47%) (Figure 2). The risk of developing T2DM after GDM increased linearly with the duration of follow-up by metaregression. The increment per year of follow-up was estimated to be 9.6‰ (95% CI, 3.6‰-15.6‰). Accordingly, when the follow-up duration extended to 10 years, 20 years, 30 years, 40 years, and 50 years, the estimated risk of T2DM was 19.72% (7.44-32.01%), 29.36% (11.07-47.65%), 39% (14.71-63.29%), 48.64% (18.35-78.92%), and 58.27% (21.99-94.56%), respectively (Figure 3).

### 3.4. Subgroup Analysis

**Figure 4:**
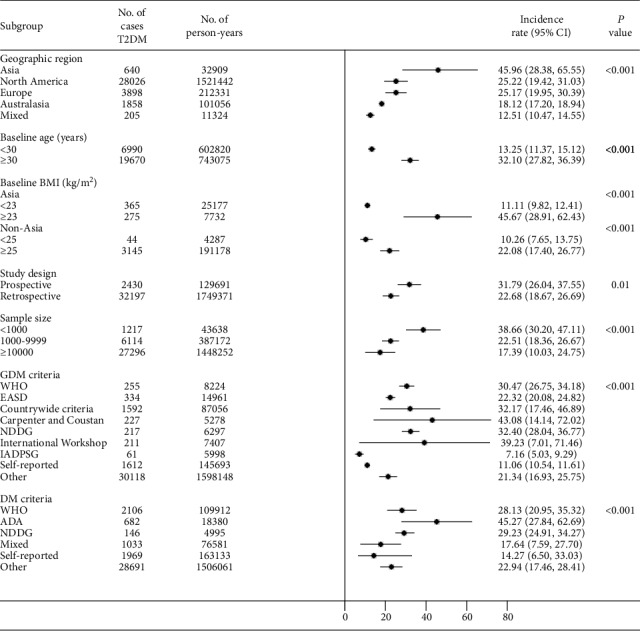


To explore the source of study heterogeneity, we performed stratified analyses across a number of key study characteristics and clinical factors, including geographic region, baseline age, baseline BMI, study design, sample sizes, diagnostic criteria for GDM, and diagnostic criteria for T2DM (Figure 4).

Differences in T2DM rates after GDM according to geographic region were statistically significant (*P* < 0.001) (Figure S1). Women from Asia had the highest incidence rate of T2DM after GDM (45.96 per 1000 person-years), followed by those from North America (25.22 per 1000 person-years), Europe (25.17 per 1000 person-years), Australasia (18.12 per 1000 person-years), and intercontinental (12.51 per 1000 person-years).

Twenty-three studies of the identified studies demonstrated the baseline age. The mean (SD) baseline age was 30.8 (2.6) years old (range from 23.9 to 35.3). The incidence rate of T2DM after GDM was significantly higher among women with age ≥ 30 years than those with age < 30 years (32.10 (95% CI, 27.82-36.39) vs. 13.25 (95% CI, 11.37-15.12) per 1000 person-years, *P* < 0.001) (Figure S2).

Fifteen studies in our meta-analysis reported a baseline BMI. The mean (SD) baseline BMI was 25.7 (2.9) kg/m^2^ (range from 20.9 to 30.9). As the average BMI of Asian women was lower than that of non-Asian women among the identified studies (23.04 kg/m^2^ vs 27.53 kg/m^2^), we conducted stratified analysis of baseline BMI by analyzing Asian and non-Asian women separately. Six studies from Asian countries and nine from non-Asian countries reported a baseline BMI. The incidence rate of T2DM after GDM was higher among Asian women whose BMI ≥ 23 kg/m^2^ (45.67 per 1000 person-years) than those with BMI < 23 kg/m^2^ (11.11 per 1000 person-years) (*P* < 0.001) (Figure S3). Similarly, the incidence rate of T2DM after GDM was significantly higher in non-Asian women with baseline BMI ≥ 25 kg/m^2^ than those with baseline BMI < 25 kg/m^2^ (22.08 vs. 10.26 per 1000 person-years, *P* < 0.001) (Figure S4).

In addition, study design and sample size might influence the results. The incidence rate of T2DM of prospective studies was 31.79 per 1000 person-years, significantly higher than 22.68 per 1000 person-years of retrospective studies (*P* = 0.01) (Figure S5). Studies with small sample size seem to reported higher incidence rate than those with large sample size (*P* < 0.001) (Figure S6).

Notably, the diagnostic criteria for GDM and T2DM in the primary studies also seem to be associated with the risk. The incidence of T2DM after GDM was highest when applying the Carpenter and Coustan (43.08) diagnostic criteria for GDM, followed by the International Workshop-Conference on GDM (39.23), NDDG (32.40), countrywide criteria (32.17), WHO (30.47), EASD (22.32), other (21.34), self-reported (11.06), and IADPSG (7.16 per 1000 person-years) (*P* < 0.001) (Figure S7). The incidence of T2DM after GDM for different diagnostic criteria for T2DM per 1000 person-years were as follows: 45.27 for ADA, 29.23 for NDDG, 28.13 for WHO, 22.94 for other criteria, 17.64 for mixed criteria, and 14.27 for self-reported (*P* < 0.001) (Figure S8).

Moreover, in one study by Cho [30], the incidence of T2DM stratified according to GDM status and the number of prepregnancy risk factors were reported. In women with or without GDM, the incidence rate of T2DM increased with the number of prepregnancy risk factors. Women with GDM had a higher incidence of T2DM than those without GDM but with the same number of prepregnancy risk factors. The incidence of T2DM was highest in women with both GDM and four prepregnancy risk factors (57.29 per 1000 person-years) and was lowest in those with neither GDM nor prepregnancy factors (3.19 per 1000 person-years) (Figure S9).

## 4. Discussion

The incidence rate of T2DM after GDM was found to be 26.20 per 1000 person-years through the present meta-analysis. The risk of developing T2DM was greater for women with GDM from Asia, with increased age, and with higher BMI in prospective studies, whereas the risk was lower when applying the IADPSG criteria to diagnose GDM. Furthermore, the risk of developing T2DM increased linearly with the duration of follow-up.

Although quite a few reviews and meta-analysis [3, 52–55] have evaluated the relative risk of T2DM after GDM, there are no available meta-analyses on the incidence rate of T2DM after GDM. This meta-analysis is the first to our knowledge to calculate the incidence rate of T2DM among women with a history of GDM.

The incidence rates of T2DM quoted in our included literatures have been extremely variable. Some characters of the studies affect the estimate rate. The highest conversion from GDM to T2DM in Asian populations in our finding might, in part, explain why Asia has emerged as the major area with a rapidly developing T2DM epidemic [56]; apart from that, Asia has the highest prevalence of GDM [57]. In Asian countries, the T2DM epidemic is characterized by onset at a lower BMI than in Western populations [58], which is also consistent with our finding. That is also why we performed subgroup analysis of baseline BMI by separating Asian and non-Asian populations and according to Asia-specific BMI cut points [59] in Asian populations. However, the reasons why Asians have a higher risk of T2DM at a lower BMI are unclear. It has been suggested that for a given BMI, Asians have a higher percentage of body fat and more visceral adipose tissue compared with other racial/ethnic groups [60]. Low BMI and a tendency toward greater abdominal obesity put Asian people at high risk of *β*-cell dysfunction and insulin resistance thus triggering T2DM [58].

Aside from populations, the large variation in the subsequent development of type 2 diabetes may also be due to the use of diverse diagnostic criteria for GDM. Diagnostic criteria for GDM have rapidly evolved during the past few decades. The IADPSG recommendations [38] are the first evidence-based, large-scale guideline established in 2010 based on glucose levels associated with adverse pregnancy outcomes in the HAPO Study [61] and in other studies. Duran et al. found the application of the new IADPSG criteria was associated with a 3.5-fold increase in GDM prevalence compared with the traditional Carpenter-Coustan criteria [62], as well as significant improvements in pregnancy outcomes. But there are few studies exploring the long-term outcomes of IADPSG-defined GDM until now. We found the incidence rate of T2DM was lowest among those with IADPSG-defined GDM (7.16 per 1000 person-years) compared with GDM defined by other diagnostic criteria. In the ATLANTIC-DIP study of white Europeans [63], 270 women with a history of GDM based on IADPSG criteria were followed up to 5 years (a mean follow-up of 2.6 years) postpartum. In total, six women demonstrated T2DM; thus, the incidence rate of 8.55 per 1000 person-years was similar to our finding. They also found the that cumulative incidence of abnormal glucose tolerance between women meeting the IADPSG criteria only and those meeting the modified WHO 1999 criteria only was of no significant differences (*P* = 0.798). It seems their findings were different from ours. However, it is uncertain, since they assessed incidence of abnormal glucose tolerance which included impaired fasting glucose, impaired glucose tolerance, and diabetes. Even if the incidence rate of T2DM after the IADPSG-defined GDM reduced, given the rising prevalence of GDM defined by IADPSG, the trend of T2DM associated with GDM is unclear which needs to be explored further.

Notably, the risk of developing T2DM after GDM tended to increase linearly with the duration of follow-up. For example, women who suffered from GDM during the age of 31 years old would have a risk of 19.72% for developing T2DM in the age 41 years old and a risk of 39% in the age 61 years old. A systematic review including 28 studies performed by Kim et al. in 2002 [53] showed that the progression to type 2 diabetes increased markedly within the first 5 years after delivery and appeared to plateau after ten years, which was not consistent with ours. The substantial differences might lie in the duration of follow-up. The follow-up time was shorter than five years among 67.86% of his studies, while 60.71% of our studies had a duration of follow-up of more than five years after GDM.

Our study has important implications. Existing reviews often accessed the relative risk of T2DM among GDM and their peers, which could not give an intuitive concept to physicians and patients. Our study provided an absolute risk of T2DM among women with GDM, which might motivate these affected mothers to attend screening programmes thus help guide lifestyle management and monitoring to reduce the future risk of T2DM.

Strengths of this meta-analysis include the strict inclusion criteria, the large number of patients analyzed, the robustness of the findings in subgroup analyses, and the relationship between follow-up duration and risk of T2DM.

Like all meta-analyses, our study has the limitation of being a retrospective analysis. Another limitation is the lack of individual participant data, which precluded stratifying results according to insulin use during pregnancy, family history of diabetes, and polycystic ovary syndrome which are the high risk factors for T2DM. However, in the study by Cho, the incidence rate of T2DM seemed to be associated with the number of prepregnancy risk factors. It suggests that multiple prepregnancy risk factors other than GDM might also increase the risk of developing T2DM. Further studies, including well-designed clinical trials, are warranted to elucidate the specific pathogenic mechanisms and the impact of other potential risk factors.

Despite these limitations, this study is, to our knowledge, the first meta-analysis to quantify the future onset risk of T2DM in women with GDM. The findings add an important insight into the trajectory of the development of type 2 diabetes in the long-term postpartum periods.

## 5. Conclusion

In conclusion, this meta-analysis provided overall estimates of T2DM incidence rates in women with previous GDM, which showed substantial differences according to geographic region, age, baseline BMI, and diagnostic criteria for GDM and T2DM. Furthermore, we found that the risk of T2DM increased linearly by 9.6‰ for every additional one year of follow-up after GDM.

## Figures and Tables

**Table 1:** Baseline characteristics of included studies.

Study	Study type	Year	Country	Geographic region	Total number of GDM	Number of T2DM	Mean follow-up (y)	Baseline age	Baseline BMI	GDM criteria	T2DM criteria
Lowe WL Jr [9]	Prospective cohort	2018	Mixed	Mixed	663	71	11.4	31.9	29.7	Carpenter and Coustan [37]^i^/IADPSG [38]^ii^	Self-reported/American Diabetes Association [39]^iii^
Shen Y [18]	Prospective cohort	2018	China	Asia	1263	121	4.4	30.1	24.2	WHO, 1999 [40]^iv^	American Diabetes Association [39]^iii^
Apostolakis M [20]	Prospective cohort	2018	Greek	Europe	1336	83	1.38	35.3	26.7	Carpenter and Coustan [37]^i^	American Diabetes Association [39]^iii^
Daly B [21]	Retrospective cohort	2018	UK	Europe	9118	895	2.9	33	Not recorded	The Health Improvement Network database^v^	The Health Improvement Network database^v^
Kugishima Y [34]	Retrospective cohort	2018	Japan	Asia	306	32	1.09	33	23.5	JSOG [41]^vi,^/IADPSG [38]^ii^	WHO, 1999 [40]^vii^
Casagrande SS [31]	Retrospective cohort	2018	USA	North America	8185	1612	17.8	23.9	Not recorded	Self-reported	Self-reported
Hippisley-Cox J [10]	Retrospective cohort	2017	England	Europe	22415	1468	3.97	Not recorded	Not recorded	QResearch database ^viii^	QResearch database ^viii^
Bond R [12]	Retrospective cohort	2017	Canada	North America	34686	6147	12.5	Not recorded	Not recorded	Quebec physician services claims; hospitalization discharge databases; and birth and death registries^ix^	Quebec physician services claims; hospitalization discharge databases; and birth and death registries^ix^
Retnakaran R [35]	Retrospective cohort	2017	Canada	North America	56884	15585	10	32	Not recorded	Hospital defined^x^	Ontario Diabetes Database [42]^xi^
Cho NH [26]	Prospective cohort	2016	Korea	Asia	412	51	3.98	30.5	23.3	Third International Workshop-Conferenceon Gestational Diabetes Mellitus [43]^xii^	American Diabetes Association [39]^iii^
Cho GJ [30]	Retrospective cohort	2016	Korea	Asia	2962	249	8	29.8	20.9	Korea National Health Insurance Claims Database^xiii^	Korea National Health Insurance Claims Database^xiii^
Wahlberg J [19]	Prospective cohort	2016	Sweden	Europe	1324	334	11.3	32	26.7	EASD [44]^xiv^	Self-reported
Gunderson EP [22]	Prospective cohort	2015	USA	North America	959	113	1.74	33.4	30	Carpenter and Coustan [37]^i^	American Diabetes Association [39]^iii^
Shen G X [13]	Retrospective cohort	2015	Canada	North America	11895	4096	30	28.8	Not recorded	ICD^xv^	Center defined^xvi^
Hakkarainen H [28]	Prospective cohort	2015	Finland	Europe	489	51	10	32	30.9	Hospital defined^xvii^	American Diabetes Association [39]^iii^
Bao W [27]	Prospective cohort	2014	UK	Europe	4554	635	13	25.5	27	Nurse's Health Study II [45]^xviii^	NDDG, 1979 [46]^xix^(before1998); ADA [39]^iii^(After 1998 June)
Kwak SH [29]	Prospective cohort	2013	Korea	Asia	395	116	3.75	31.4	22.8	Third International Workshop-Conference on Gestational Diabetes Mellitus [43]^xii^	American Diabetes Association [39]^iii^
Ekelund M [15]	Retrospective cohort	2012	Mixed	Mixed	793	134	4.75	Not recorded	Not recorded	WHO, 1999 [40]^iv^	WHO, 1999 [40]^vii^
Ziegler AG [25]	Prospective cohort	2012	Germany	Europe	304	147	7.9	31	Not recorded	German Diabetes Association^xx^	American Diabetes Association [39]^iii^
Wang YJ [33]	Retrospective cohort	2012	USA	North America	1142	327	8.6	26.8	Not recorded	ICD^xxi^	American Diabetes Association [39]^iii^ /WHO, 1998 [47]^xxii^
Anderberg E [36]	Retrospective cohort	2012	Sweden	Europe	579	180	11	Not recorded	Not recorded	Diagnostic registers from hospital^xxiii^	ICD^xxiv^
Kakad R [14]	Retrospective cohort	2010	UK	Europe	470	38	1	Not recorded	Not recorded	Hospital defined^xxv^	WHO^xxvi^
Lee H [32]	Prospective cohort	2008	Korea	Asia	620	71	2.1	33.6	23.5	National Diabetes Data Group, 1979 [46]^xxvii^	Hospital defined^xxviii^
LEE A J [17]	Retrospective cohort	2007	Australia	Australia	5470	1411	15	30.7	26.9	Australia guideline [48]^xxix^	WHO, 1998 [47]^xxii^
Järvelä IY [16]	Retrospective cohort	2006	Finland	Europe	435	23	5.7	31.6	Not recorded	Finnish Diabetes Association^xxx^	Self-reported
Albareda M [11]	Prospective cohort	2003	Spain	Europe	696	44	6.16	30.7	24.5	Second and Third GDM Workshop Conference [49]^xxxi^	WHO, 1998 [47]^xxii^
Peters RK [23]	Prospective cohort	1996	USA	North America	666	146	7.5	30.3	Not recorded	National Diabetes Data Group, 1979 [46]^xxvii^	National Diabetes Data Group, 1979 [46]^xix^
Henry O [24]	Prospective cohort	1991	Australia	Australia	1118	447	17	31.3	25.4	Hospital defined [50]^xxxii^	WHO, 1985 [51]^xxxiii^

FPG: fasting plasma glucose. OGTT: oral glucose tolerance test. ICD: International Classification of Disease. Baseline age: the age at index pregnancy. Baseline BMI: the BMI at the beginning of follow-up. i: FPG concentrations ≥ 5.3 mmol/L, 2 h plasma glucose ≥ 8.6 mmol/L, or 3 h glucose ≥ 7.7 mmol/L after 100 g oral glucose load. ii: FPG concentrations ≥ 5.1 mmol/L, 1 h plasma glucose ≥ 10.0 mmol/L, or 2 h glucose ≥ 8.5 mmol/L after 75 g oral glucose load. iii: FPG concentrations ≥ 7.0 mmol/L or 2 h glucose ≥ 11.1 mmol/L after 75 g oral glucose load. iv: if 1 h plasma glucose is > 7.8 mmol/L after 50 oral glucose load, then 75 g glucose test is performed, FPG concentrations ≥ 7.0 mmol/L or 2 h glucose ≥ 11.1 mmol/L after 75 g oral glucose load. v: the database captures electronically recorded medical records in primary care. vi: FPG concentrations ≥ 5.6 mmol/L, 1 h plasma glucose ≥ 10.0 mmol/L, or 2 h glucose ≥ 8.3 mmol/L after 75 g oral glucose load, two or more abnormal values. vii: FPG concentrations ≥ 7.0 mmol/L or 2 h glucose ≥ 11.1 mmol/L after 75 g oral glucose load. viii: identify patients with diabetes by searching the electronic health record for diagnostic Read codes for diabetes (C10%). ix: ICD 9 and 10 codes are used to identify diagnoses of gestational diabetes. x: diagnostic codes associate with the delivery hospitalization. xi: all individuals, living within Ontario, Canada, diagnosed with diabetes ≥90 days after delivery were included. xii: FPG ≥ 5.8 mmol/L, 1 h glucose ≥ 10.6 mmol/L, 2 h glucose ≥ 9.2 mmol/L, and 3 h glucose ≥ 8.1 mmol/L after 100 g glucose load, two or more abnormal values. xiii: the ICD 10^th^ revision codes O24.4 and O24.9 are used to identify diagnoses of gestational diabetes, and the ICD 10^th^ revision code E11 is used to identify diagnoses of diabetes. xiv: 2 h capillary blood glucose ≥ 9.0 mmol/L after 75 g OGTT. xv: ICD-10-CA codes were used after 1 April 2004 or ICD-9-CM code prior to that date to identify the diagnoses of gestational diabetes. xvi: incident diabetes among women are defined as one hospitalization or two physicians' diagnoses of diabetes within a 3-year period. xvii: until September 2001: lower abnormal limits of fasting, 1 h and 2 h capillary whole-blood glucose are 4.8, 10.0, and 8.7 mmol/L; September 2001: lower limits of fasting, 1 h and 2 h capillary plasma glucose are 4.8, 11.2, and 9.9 mmol/L. xviii: 116,671 female nurses aged 25 to 44 years were included at study initiation. xix: FPG ≥ 7.8 mmol/L or 2 h glucose ≥ 11.1 mmol/L after 75 g OGTT. xx: two of three capillary blood glucose values > 5 mmol/L (fasting) before OGTT; 1 h OGTT ≥ 10.6 mmol/L and 2 h OGTT ≥ 8.9 mmol/L. xxi: ICD-9 code 648.8. xxii: FPG ≥ 7.8 mmol/L (≥7.0 mmol/L from 1998), 2 h glucose ≥ 11.1 mmol/L after 75 g OGTT, and one or more classic symptoms plus a random plasma glucose level ≥ 11.1 mmol/L. xxiii: identified through diagnostic registers from the University Hospitals of Lund and Malmö. xxiv: the ICD 10^th^ revision codes E10 or E11 were used to identify the diagnoses of diabetes. xxv: use 75 g OGTT to diagnose gestational diabetes. xxvi: FPG ≥ 7.1 mmol/L and/or 2 h plasma glucose ≥ 11.0 mmol/L after 75 g glucose load. xxvii: requires 2 abnormal glucose values: FPG ≥ 5.8 mmol/L, 1 h glucose ≥ 10.6 mmol/L, 2 h glucose ≥ 9.2 mmol/L, and 3 h glucose ≥ 8.1 mmol/L. xxviii: FPG ≥ 7.0. xxix: after 1999.1.1: FPG ≥ 5.5 mmol/L and/or 2 h glucose ≥ 8.0 mmol/L after 75 g OGTT; before 1999.1.1: 1 h capillary plasma glucose level of ≥9.0 mmol/L and a 2 h capillary plasma glucose level of ≥7.0 mmol/L after 50 g OGTT. xxx: the limits of abnormal capillary blood glucose concentrations used are as follows: fasting ≥ 4.8 mmol/L, 1 h ≥ 10 mmol/L, and 2 h ≥ 8.7 mmol/L after 75 g OGTT. xxxi: FPG ≥ 5.8 mmol/L, 1 h glucose ≥ 10.6 mmol/L, 2 h glucose ≥ 9.2 mmol/L, and 3 h glucose ≥ 8.1 mmol/L after 100 g glucose load, two or more abnormal values. xxxii: 1 h glucose level ≥ 9.0 mmol/L and 2 h glucose level ≥ 7.0 mmol/L after 50 g OGTT. xxxiii: capillary plasma glucose: fasting ≥ 7.8 mmol/L or 2 h ≥ 12.2 mmol/L after 75 g OGTT.

## Data Availability

The data used to support the findings of this study are available from corresponding author upon request.

## Supplementary Materials

Supplementary Materials Figure S1: forest plot of incidence rates of T2DM after GDM per 1000 person-years of follow-up stratified by geographic region. Horizontal lines indicate 95% CIs. Differences in T2DM rates after GDM according to geographic region were statistically significant (*P* < 0.001). Figure S2: forest plot of incidence rates of T2DM after GDM per 1000 person-years of follow-up stratified by baseline age. Horizontal lines indicate 95% CIs. The incidence rate of T2DM after GDM was significantly higher among women with age ≥ 30 years than those with age < 30 years (*P* < 0.001). Figure S3: forest plot of incidence rates of T2DM after GDM per 1000 person-years of follow-up stratified by BMI of Asian. Horizontal lines indicate 95% CIs. The incidence rate of T2DM after GDM was higher among Asian women whose BMI ≥ 23 kg/m^2^ than those with BMI < 23 kg/m^2^ (*P* < 0.001). Figure S4: forest plot of incidence rates of T2DM after GDM per 1000 person-years of follow-up stratified by BMI of non-Asian. Horizontal lines indicate 95% CIs. The incidence rate of T2DM after GDM was significantly higher in non-Asian women with baseline BMI ≥ 25 kg/m^2^ than those with baseline BMI < 25 kg/m^2^ (*P* < 0.001). Figure S5: forest plot of incidence rates of T2DM after GDM per 1000 person-years of follow-up stratified by study design. Horizontal lines indicate 95% CIs. The incidence rate of T2DM of prospective studies was significantly higher than retrospective studies (*P* = 0.01). Figure S6: forest plot of incidence rates of T2DM after GDM per 1000 person-years of follow-up stratified by sample size of GDM. Horizontal lines indicate 95% CIs. Studies with small sample size reported higher incidence rate than those with large sample size (*P* = 0.01). Figure S7: forest plot of incidence rates of T2DM after GDM per 1000 person-years of follow-up stratified by GDM criteria. Horizontal lines indicate 95% CIs. The incidence of T2DM after GDM was highest when applying the Carpenter and Coustan (43.08) diagnostic criteria for GDM and was lowest when applying IADPSG (7.16 per 1000 person-years) (*P* < 0.001). Figure S8: forest plot of incidence rates of T2DM after GDM per 1000 person-years of follow-up stratified by T2DM criteria. Horizontal lines indicate 95% CIs. The incidence of T2DM after GDM for different diagnostic criteria for T2DM per 1000 person-years was significantly different (*P* < 0.001). Figure S9: incidence rate of type 2 diabetes stratified by GDM status and the number of prepregnancy risk factors according to the study of Cho [30].
