# Supplementary material for: Incidence Rate of Type 2 Diabetes Mellitus after Gestational Diabetes Mellitus: A Systematic Review and Meta-Analysis of 170,139 Women
Source: J Diabetes Res. 2020 Apr 27;2020:3076463. doi: 10.1155/2020/3076463 (PMC7204113; doi:10.1155/2020/3076463)
Supplement: Supplementary Materials — Figure S1: forest plot of incidence rates of T2DM after GDM per 1000 person-years of follow-up stratified by geographic region. Horizontal lines indicate 95% CIs. Differences in T2DM rates after GDM according to geographic region were statistically significant (P < 0.001). Figure S2: forest plot of incidence rates of T2DM after GDM per 1000 person-years of follow-up stratified by baseline age. Horizontal lines indicate 95% CIs. The incidence rate of T2DM after GDM was significantly higher among women with age ≥ 30 years than those with age < 30 years (P < 0.001). Figure S3: forest plot of incidence rates of T2DM after GDM per 1000 person-years of follow-up stratified by BMI of Asian. Horizontal lines indicate 95% CIs. The incidence rate of T2DM after GDM was higher among Asian women whose BMI ≥ 23 kg/m2 than those with BMI < 23 kg/m2 (P < 0.001). Figure S4: forest plot of incidence rates of T2DM after GDM per 1000 person-years of follow-up stratified by BMI of non-Asian. Horizontal lines indicate 95% CIs. The incidence rate of T2DM after GDM was significantly higher in non-Asian women with baseline BMI ≥ 25 kg/m2 than those with baseline BMI < 25 kg/m2 (P < 0.001). Figure S5: forest plot of incidence rates of T2DM after GDM per 1000 person-years of follow-up stratified by study design. Horizontal lines indicate 95% CIs. The incidence rate of T2DM of prospective studies was significantly higher than retrospective studies (P = 0.01). Figure S6: forest plot of incidence rates of T2DM after GDM per 1000 person-years of follow-up stratified by sample size of GDM. Horizontal lines indicate 95% CIs. Studies with small sample size reported higher incidence rate than those with large sample size (P = 0.01). Figure S7: forest plot of incidence rates of T2DM after GDM per 1000 person-years of follow-up stratified by GDM criteria. Horizontal lines indicate 95% CIs. The incidence of T2DM after GDM was highest when applying the Carpenter and Coustan (43.08) diagnostic criteria fo [file 3076463.f1.docx]

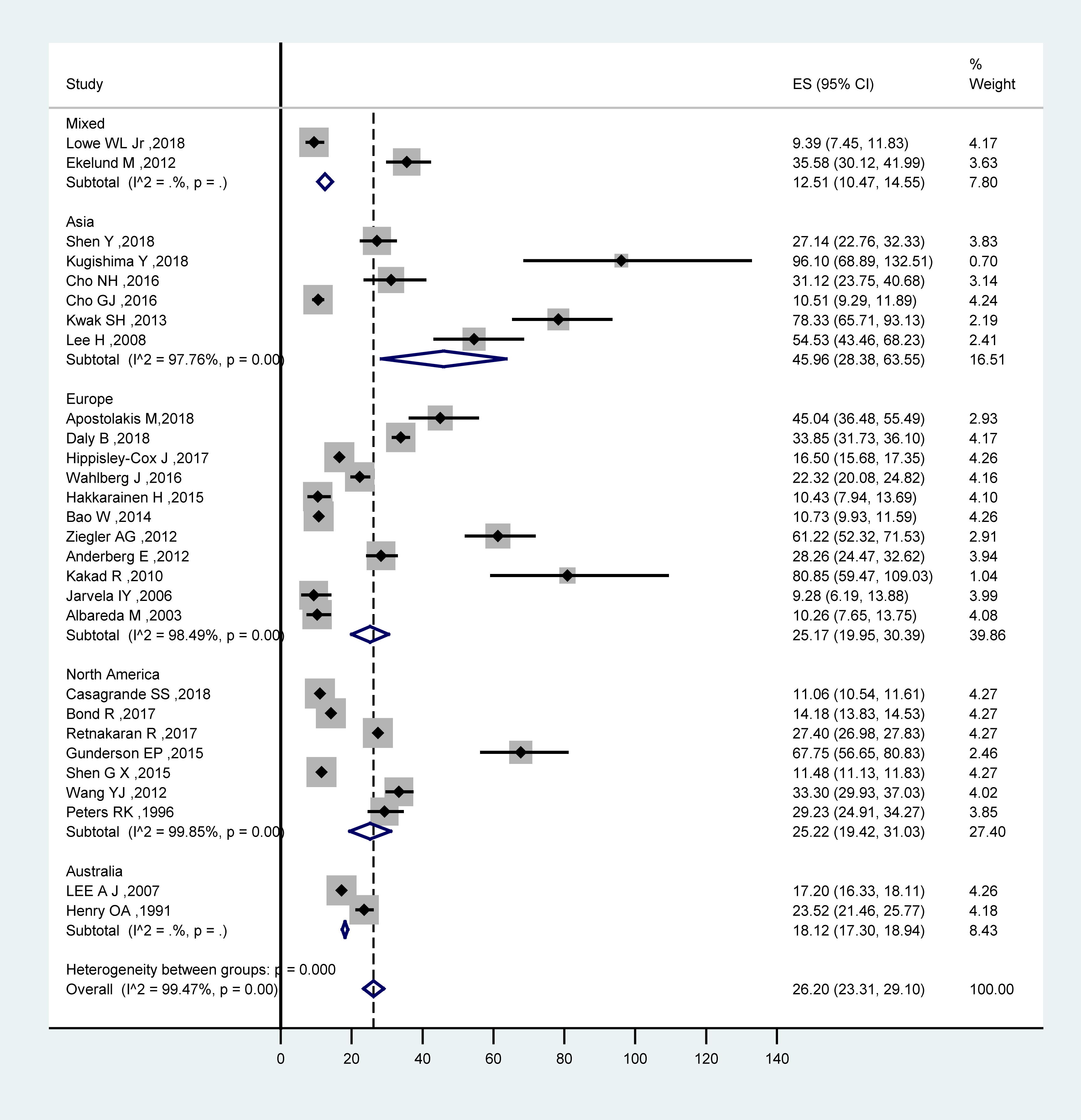


**Figure S1:** **Forest plot of incidence rates of T2DM after GDM per 1000 person-years of follow-up stratified by geographic region.** Horizontal lines indicate 95% CIs. Differences in T2DM rates after GDM according to geographic region were statistically significant (*P*<0.001)


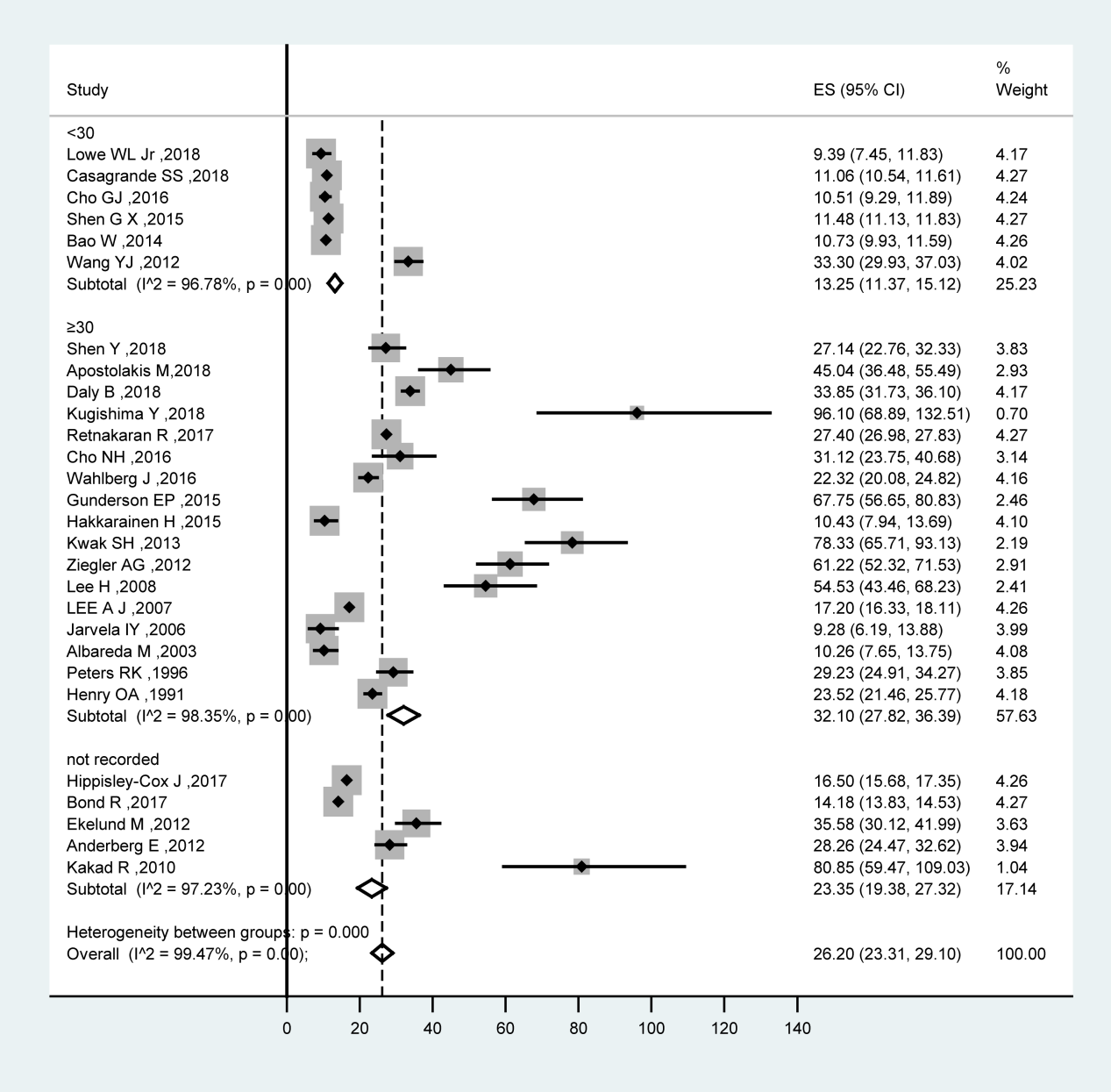


**Figure S2:** **Forest plot of incidence rates of T2DM after GDM per 1000 person-years of follow-up stratified by baseline age.** Horizontal lines indicate 95% CIs. The incidence rate of T2DM after GDM was significantly higher among women with age≥30 years than those with age<30 years(*P*<0.001).

**
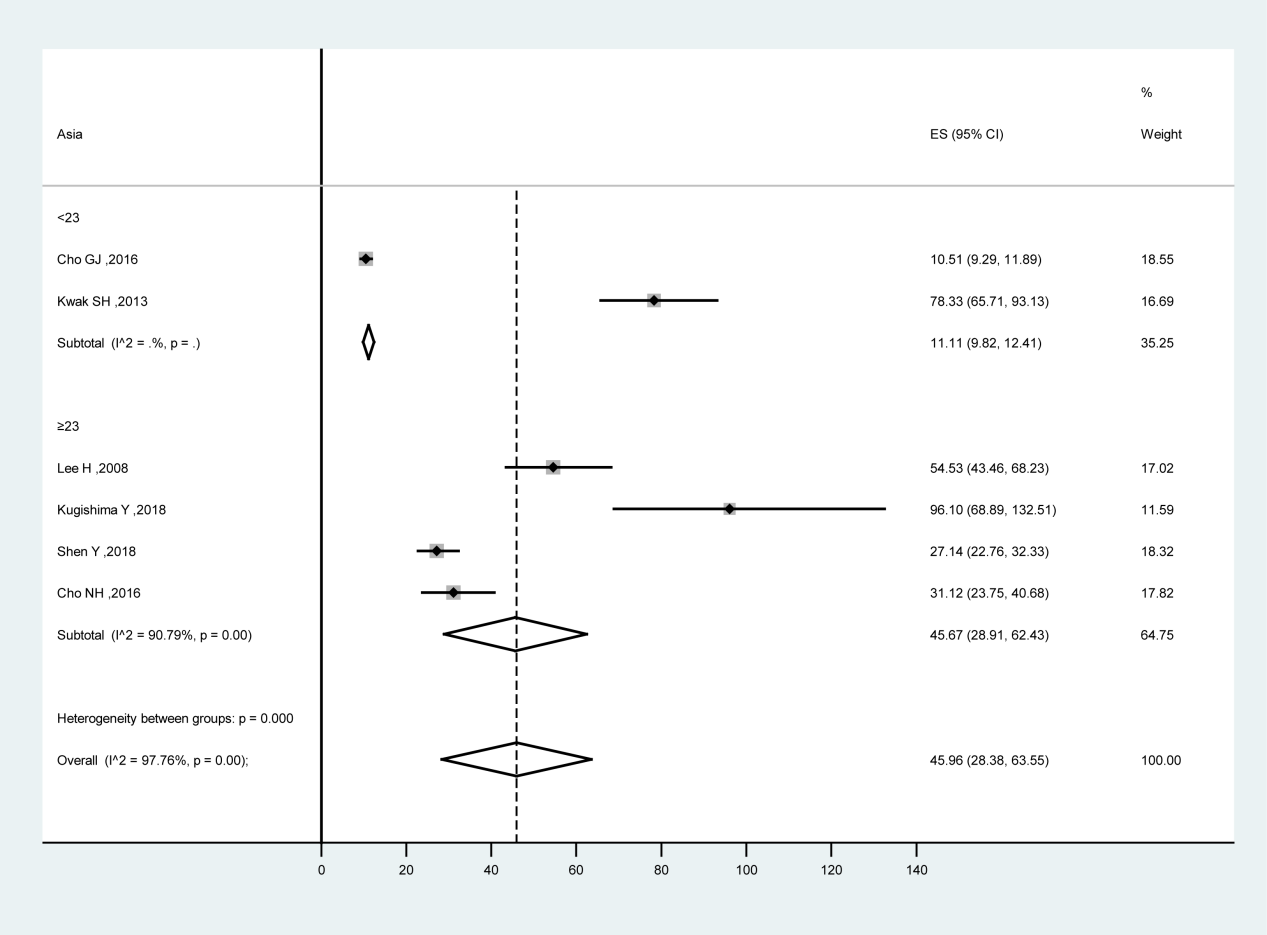
**

**Figure S3:** **Forest plot of incidence rates of T2DM after GDM per 1000 person-years of follow-up stratified by BMI of Asian.** Horizontal lines indicate 95% CIs. The incidence rate of T2DM after GDM was higher among Asian women whose BMI≥23 kg/m^2^ than those with BMI<23 kg/m^2^ (*P*<0.001).

**
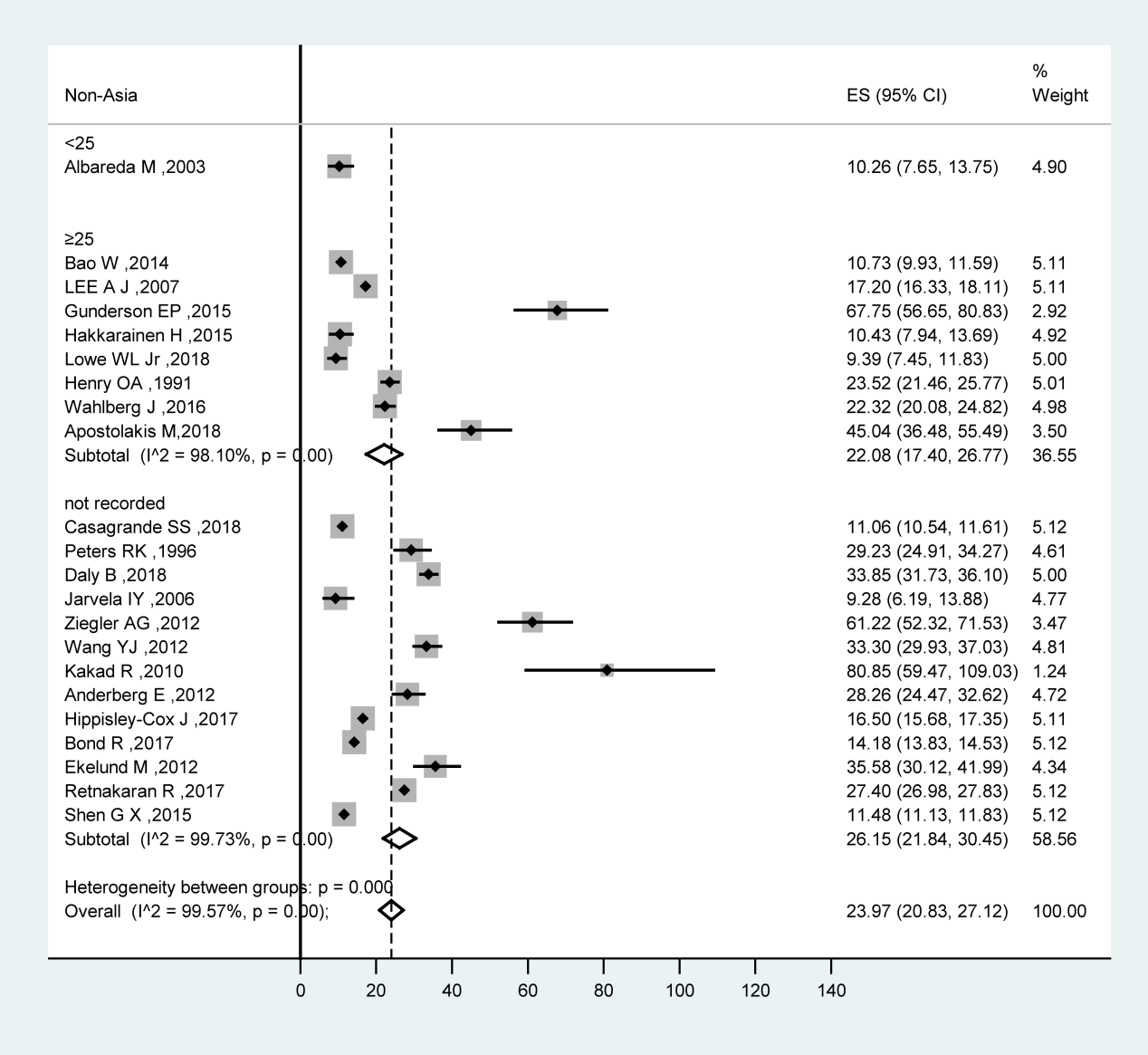
**

**Figure S4:** **Forest plot of incidence rates of T2DM after GDM per 1000 person-years of follow-up stratified by BMI of non-Asian.** Horizontal lines indicate 95% CIs. The incidence rate of T2DM after GDM was significantly higher in non-Asian women with baseline BMI≥25 kg/m^2^ than those with baseline BMI<25 kg/m^2^ (*P*<0.001).

**
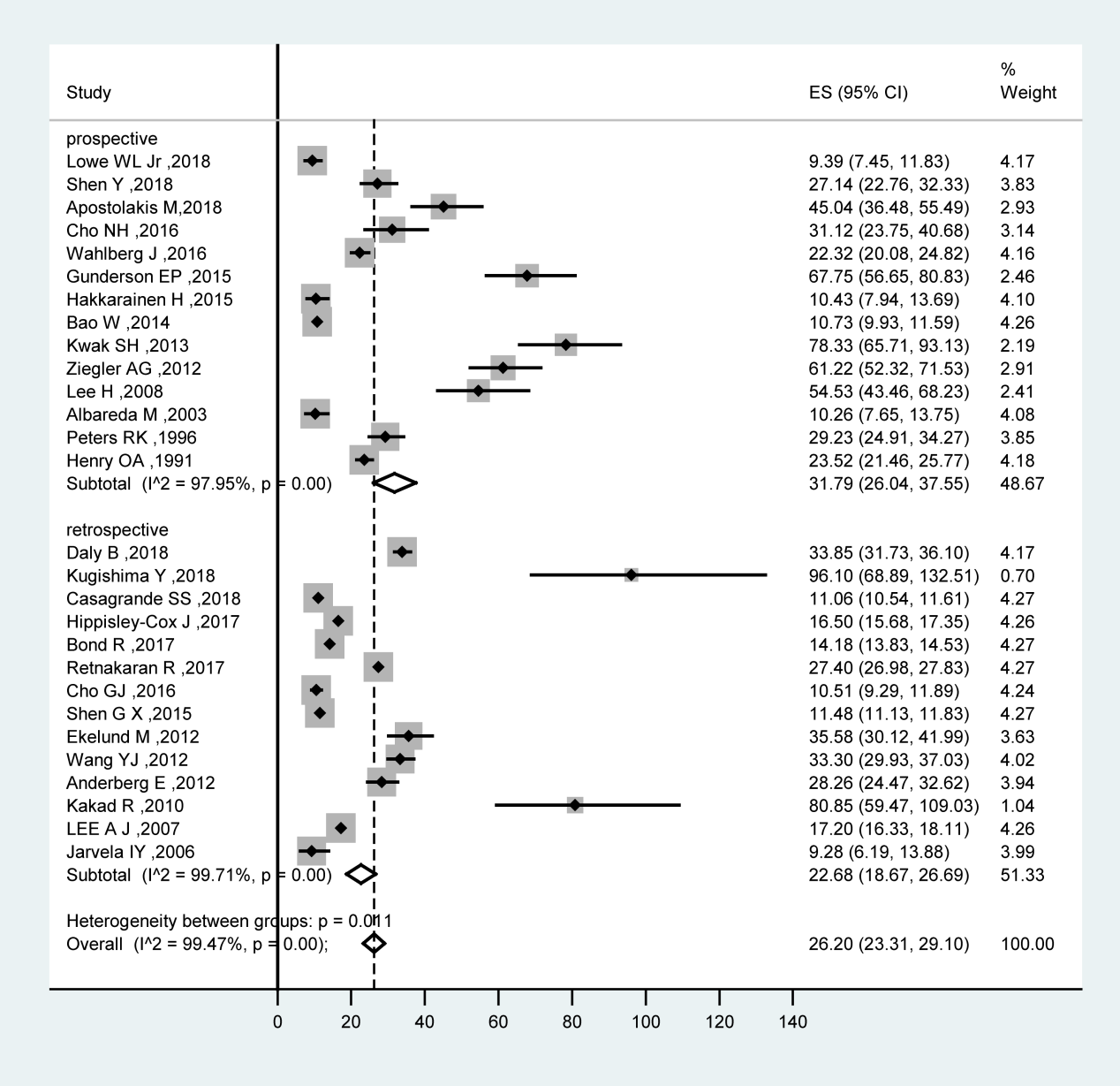
**

**Figure S5:** **Forest plot of incidence rates of T2DM after GDM per 1000 person-years of follow-up stratified by study design.**Horizontal lines indicate 95% CIs. The incidence rate of T2DM of prospective studies was significantly higher than retrospective studies (*P*=0.01).

**
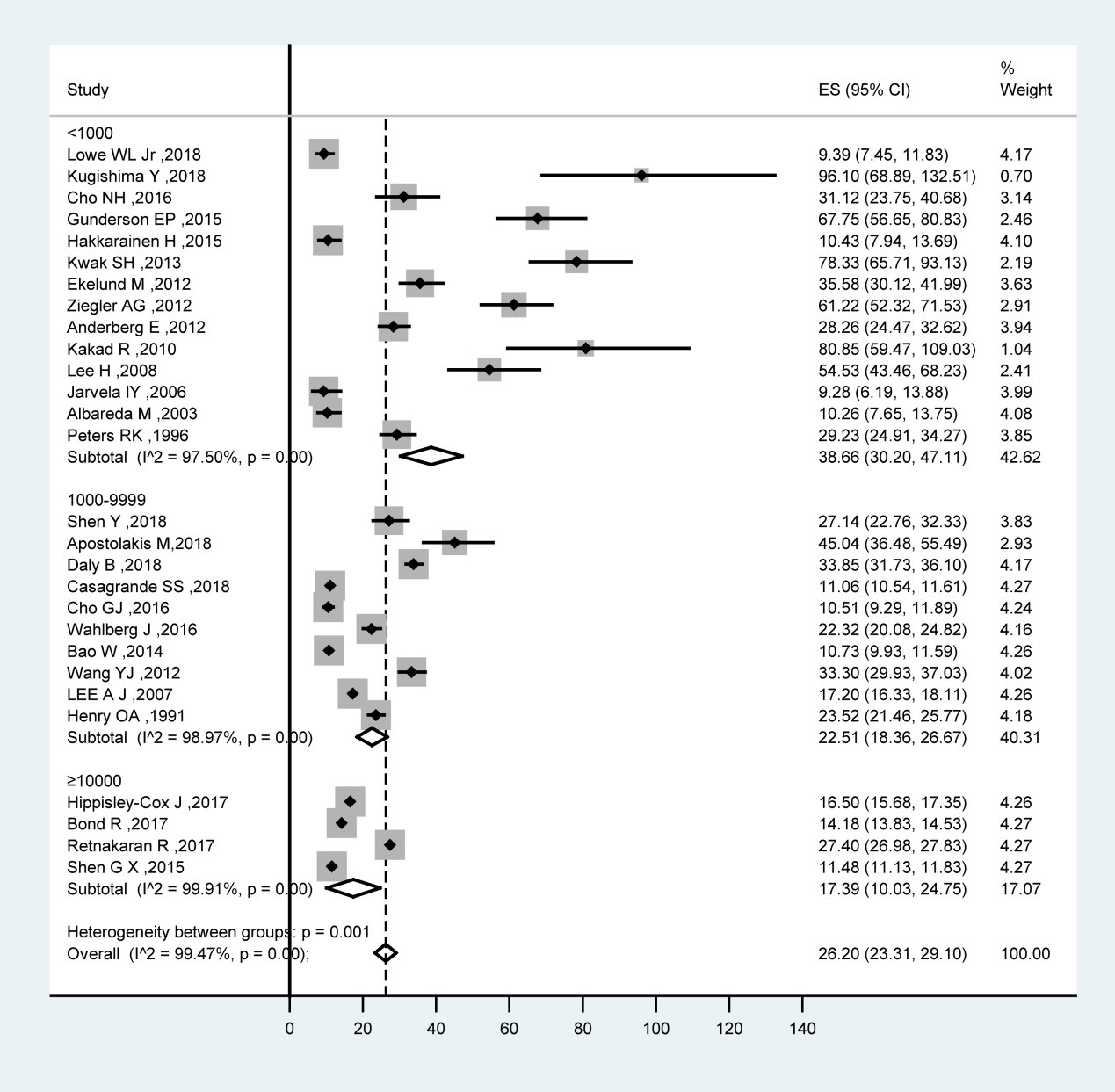
**

**Figure S6:** **Forest plot of incidence rates of T2DM after GDM per 1000 person-years of follow-up stratified by sample size of GDM.** Horizontal lines indicate 95% CIs. Studies with small sample size reported higher incidence rate than those with large sample size(*P*=0.01).

**
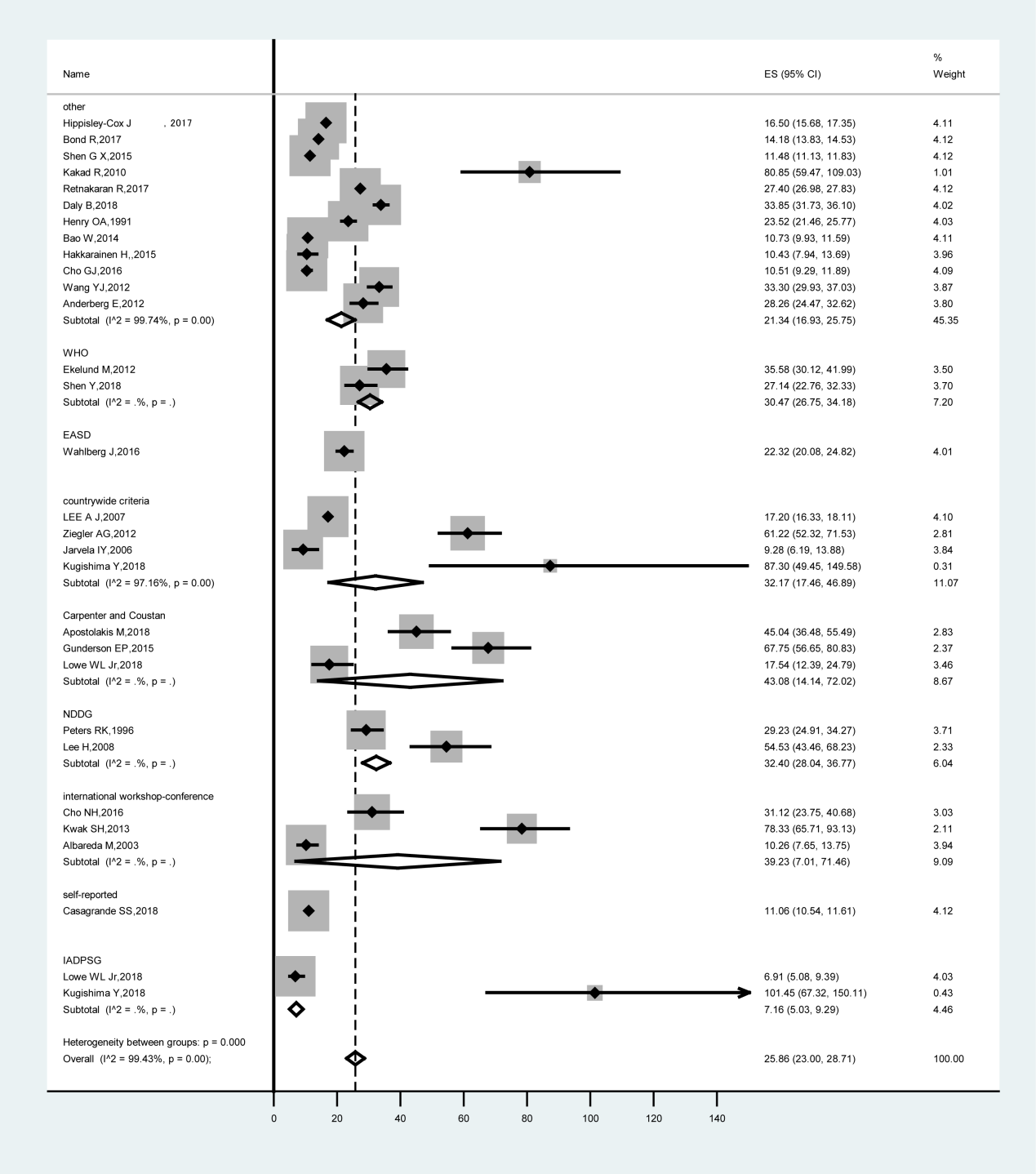
 Figure S7:** **Forest plot of incidence rates of T2DM after GDM per 1000 person-years of follow-up stratified by GDM criteria.** Horizontal lines indicate 95% CIs. The incidence of T2DM after GDM was highest when applying Carpenter and Coustan (43.08) diagnostic criteria for GDM and was lowest when applying IADPSG (7.16 per 1000 person-years) (*P*<0.001).

**
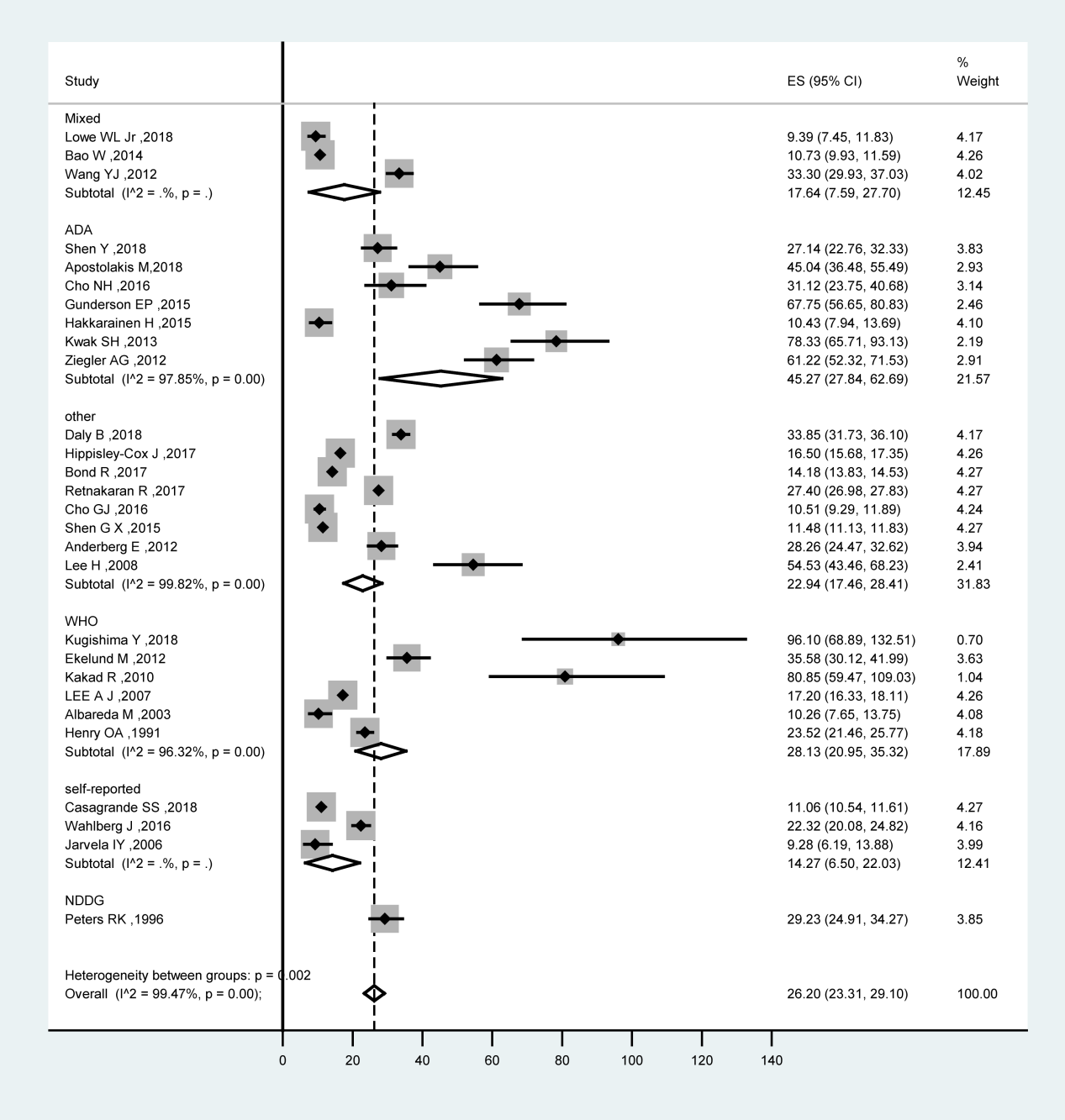
Figure S8:** **Forest plot of incidence rates of T2DM after GDM per 1000 person-years of follow-up stratified by T2DM criteria.** Horizontal lines indicate 95% CIs. The incidence of T2DM after GDM for different diagnostic criteria for T2DM per 1000 person-years were significantly different (*P*<0.001).


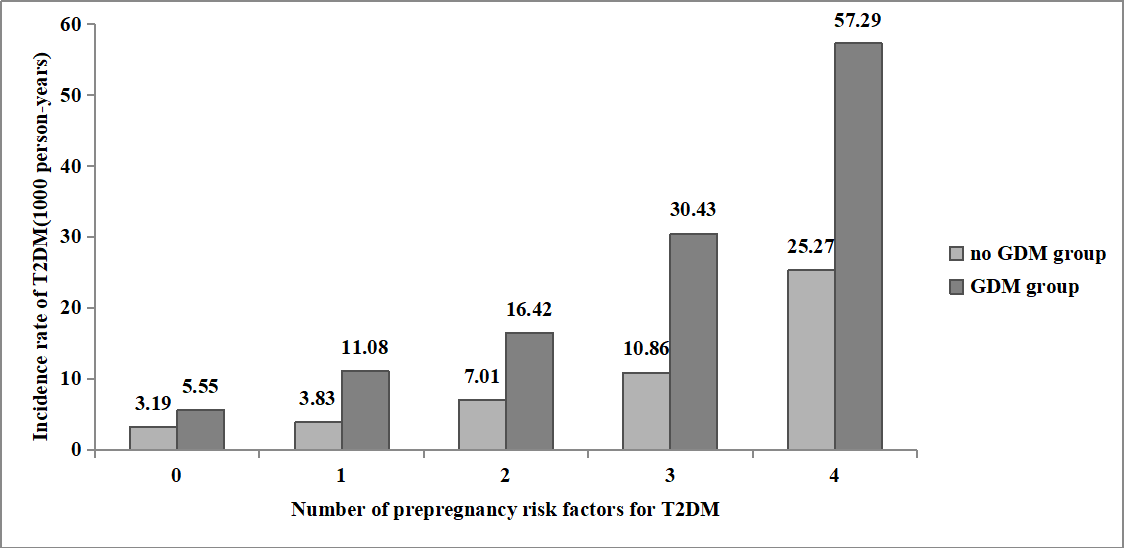


**Figure S9: Incidence rate of type 2 diabetes stratified by GDM status and the number of prepregnancy risk factors according to the study of Cho GJ[30].**
